# Operative Treatment of Knee Joint Disease

**Published:** 1893-12

**Authors:** De Forest Willard

**Affiliations:** Clinical Professor of Orthopaedic Surgery, University of Pennsylvania, Surgeon to the Presbyterian Hospital, Etc., Philadelphia, Pa.


### Southern Medical Record.

A MONTHLY JOURNAL OF MEDICINE AND SURGERY.

VOL. XXIII. ATLANTA, GA., DECEMBER, 1893. No. 12.

### Original Articles.

### OPERATIVE TREATMENT OF KNEE JOINT DISEASES.

BY DE FOREST WILLARD, M. D.,

Clinical Professor of Orthopaedic Surgery, University of Pennsylvania,
Surgeon to the Presbyterian Hospital, Etc., Philadelphia, Pa.

It is hardly necessary to state that the mechanical treatment of knee joint diseases is an essential condition to success, both before and after operative measures. This mechanical protection of the joints is to be given in the very inception of the disease, and is one of the surest preventives of suppuration, in a great majority of cases superceding all necessity for operation. These mechanical measures should include absolute rest, protection and fixation of the joint, with the avoidance of weight bearing upon the limb by the use of crutches, either axillary, perineal or ischiatic.

A large number of neglected cases, however, will demand some form of operative relief.

The operative treatment of knee joint diseases may be divided into:

I.—Tenotomy, with fixation.

II.—Removal of serum.

III.—Removal of pus accumulations and degenerated tissues inside and outside the joint.

*a*—Aspiration.

*b*—Incision and drainage.

*c*—Erasion.

*d*—Excision.

*e*—Amputation.

IV.—Hypodermic injections of anti-bacillary substances, as iodoform, chloride of zinc, etc.

V.—Removal of late deformity.

*a*—Tenomoty.

*b*—Forcible extension in tenomoty.

*c*—Osteotomy.

*d*—Partial or complete excision.

VI.—Treatment of posterior displacement.

I.—Tenotomy with fixation.

In children under ten years of age, in fact, under twelve years, it is especially important to preserve the epiphyseal line as long as possible, in order to secure the greatest possible growth of the limb; conservative surgery, therefore, in this class of cases, is to be carried to the extreme degree of mechanical treatment, and this should be thoroughly insisted upon. Operative measures, when necessary, should also be conservative in character, advancing from the milder to the more extreme procedures only when imperatively demanded. In the majority of cases, even when marked flexion exists, and when evidences of induration about the joint are positive, it is wise to temporize, improving the position of the limb by tenotomy of the ham string tendons and securing absolute immobility by plaster of Paris. By these means many cases can be cured, and even though they advance ultimately to suppuration much time is thereby gained with benefit of growth.

The question of erasion will be studied under another heading, but radical measures in these young children are especially to be deplored.

II.—Removal of Serum.

The prevention of synovial effusion consists first in subdueing the inflammation by absolute rest in bed, a posterior splint slightly flexed at the knee, ice bags or the continual dripping of iced water and by other antiplogistic remedies. Presuming that absorbents, blisters and counter irritants to produce absorption have proven unavailing, the question of tapping the joint should be considered. It is important to do this before suppuration takes place.

Aspiration for serus effusion, if performed thoroughly antiseptically, rarely gives trouble; yet inflammatory conditions, and even death, have followed this operation. An aspirating trocar of medium size may be carried into the anterior part of the joint. Should the fluid be clear, rest and fixation should be continued, and the patient kept recumbent with a pulley extension weight upon the limb. Should reaccumulation take place, the operation may be repeated as long as only serum is drawn. When pus is definitely determined, or when the symptoms point to its formation, other methods should be considered.

In the acute cases, such as are above described, the diagnosis of formation of pus is not difficult; but in chronic cases, which are slow in progress, and probably tubercular in character, where there is great thickening about the articulation, and probably much induration, it is not always easy to determine whether the deposit is inflammatory or purulent, or whether it is the thickened and gelatinous degeneration which so frequently occurs in tuberculosis.

If the deposit is of the pulpy variety, the area will be doughy and boggy. All the anatomical features of the joint will be destroyed, and the fossae upon either side of the petella obliterated. Decided flexion will exist. Pain may not be present, and in some cases this destructive process may have advanced so far that the lateral ligaments have undergone pulpy degeneration, and partial dislocation of the tibia may have taken place. When the above symptoms are present, there is but little doubt that pulpy degeneration is present, and that operative procedures are necessary. The temperature chart and symtomatic conditions will, of course, assist in diagnosis.

III.—Removal of Pus.

*a*—Aspiration is of but little permanent service in the removal of pus accumulations except for diagnostic purposes.

*b*—Incision and Drainage.

Incision into a knee is usually for the purpose of exploration, and the surgeon, previous to etherization, should have obtained the permission of the patient, or of his friends, to go forward with whatever operation may be advisable; usually it will be erasion or excision.

Simple incision is of but little service even in cases of purulent synovitis, which are acute in character. In tubercular cases, if the more radical operation is refused by the patient, of course incision permits the introduction directly into the walls of the abscess of thoroughly aseptic and anti-bacillary substances, such as iodoform, chloride of zinc, and the withdrawal of pus, which proceedings faciliate the destruction of the bacilli. As a rule, however, these can be introduced subcutaneously into the deposit.

When an incision has been made and pus found, it will, of course, be followed by drainage of the parts. This will be done either with rubber tubes or with gauze packing, after syringing of the parts with per oxide of hydrogen, bi-chloride of zinc or iodoform oil. Usually the wound, if packed for forty-eight hours and then closed by sutures inserted at the time of operation, will heal kindly. If rubber drainage is used it should not be allowed to remain long in position, since a sinus will establish itself as the tube is slowly withdrawn. The mouth of the sinus can be kept open by a small piece of tube as long as it is desirable. Of course, throughout the whole treatment, most rigid attention must be given to rest and fixation of the articulation. Without this adjunct all operative treatment will fail. The great difficulty is to keep the wound continually aseptic. Care should be taken to allow as much iodoform oil as possible to remain in the joint at each dressing. When the discharge is long continued, and the patient refuses further operation, the antiseptics should be changed every three weeks, as the tissues become accustomed to their use.

*c*—Erasion.

What class of cases are suitable for erasion and what should be reserved for excision?

These are exceedingly important questions, and the answer is frequently impossible to be determined until the exploatory incision has been made.

Erasion is especially indicated in the pulpy form of degeneration; in cases where the involvement of the articulation itself is greater than that of the bone, in children under twelve years of age, where there is a prospect that by adopting this measure shortening of the limb will be prevented, and in cases where the foci of disease are few and small. In all these cases removal of the diseased hard and soft parts may be performed with confidence, and most excellent results will be obtained.

To be of service the operation of erasion must be very thorough in its character. Every particle of degenerative material must be removed, and the knife, spoon and gouge must be applied with vigor. The prolongation of the synovial membrane above and below the patella, wherever involved, must be taken away. When such a degree of thorough removal is possible, we may confidently hope for cessation of the disease, and even if the limb is anchylosed it will be of great service to the individual, since it will be but slightly shortened. In my experience relapses are less frequent when anchylosis in the straight position has taken place than when motion has been secured.

Every care must be taken by frequent douching during the operation to prevent infection of the surrounding tissue, and especially by the use of the hollow flushing gouge.

There are two methods of dressing such a wound; the preferable one is by thoroughly packing with iodoform gauze and then closing with previously inserted sutures at the first dressing. Drainage tubes may be employed in cases where it is uncertain that the entire deposit has been removed. The skin wound is closed except at the points of exit of the drainage tubes, and the cavity thoroughly washed with iodoform oil (10 per cent.) at each dressing, after washing with bichloride and with peroxide of hydrogen, in order to guard against infection.

*d*—Excision.

When there has been prolonged suppuration, or where there are evidences of a large amount of dead bone, or where the patient has suffered from prolonged drain, especially about the age of puberty and adults, excision becomes imperative. Of course it is resorted to with much less frequency than in former years since the introduction of erasion; but in the cases above alluded to, it is an operation of extreme value. In children under ten years of age it is always well to avoid interference with the epiphyseal growth as long as possible, in order to prevent the great shortening of the limb so frequently seen when early excision is performed. Tenotomy and fixation, or eversion, as already described, will often susceed in preventing this more mutilatory operation.

If excision is determined upon the operation should be thorough, but it is not always necessary to remove the entire diameter of the bone to a point as high as the extreme foci of disease. These foci may be gouged or scraped out, thus avoiding the extreme shortening which occurs when large excisions are made.

There have been different incisions proposed for this operation. I find nothing better than the ordinary horse shoe cut, with convexity upward or downward, with division of the tendo patella. The incision should commence well upon the side of the condyles, to permit free inspection of the joint, which also faciliates drainage, and is in every way satisfactory. The straight incision, with sawing of the patella, I do not like, as union of the bone is no more probable than union of the tendo patella, if the latter is carefully sutured. Very frequently the patella has to be removed, and for removal the straight incision offers no advantages over the curved one.

The question of drainage in excision of the knee must depend upon the judgment of the surgeon. If the wound has been thoroughly cleared and thoroughly dried, the drainage may be omitted, the gauze packing before described being employed. In the majority of old suppurative cases, however, it is better to introduce a tube, at least for a few days.

The use of an Esmarch bandage renders the area of operation bloodless, and thus greater opportunity is secured for removal of all the diseased tissue; but it certainly favors subsequent oozing. Filling a wound with a bloof clot, as advocated by many surgeons, I have not found to hasten union.

The question of fixation of the excised ends has been the subject of much controversy. The use of ivory or steel nails, wiring, kangaroo tendon or silk sutures, and various other methods have been advised. For myself, I prefer to depend upon the external splint entirely, believing that the methods mentioned do not favor union, and that their only recommendation is to secure rest of the parts. This can be done by other means; therefore, I apply immediately a plaster of Paris cast. Plaster of Paris necessarily becomes soiled by the oozing of blood and serum, but in itself it is so much of an antiseptic that will remain sweet for a long time, and decomposition will not take place for a number of weeks. When removal is necessary the splint can be slit into anterior and posterior halves by sawing down either side, and the cap can be removed without disturbance of the limb.

When partial healing has taken place a second cast can be applied, made strong anteriorly, through which a large lateral and posterior window can be cut for dressing purposes.

Arched iron strips surrounding the joint, and fixed by wide gypsum enclosures of thigh and leg, are convenient for subsequent dressing. but do not hold the joint as perfectly in position as does the simple encasement of plaster of Paris. The same may be said of the bracketed splint so frequently employed.

*e*—Amputation.

The removal of the limb in adults is in many cases of extensive diseased joints, the wisest procedure. In any case of doubt, an exploatory incision into the joint gives the opportunity either for an excision or an amputation.

IV.—Hypodermic Injections of Anti-Bacillary Substances.

My experience with this method has not extended beyond fifty cases, too small a number to draw any conclusions of value. I am still testing it carefully, and while I have had excellent results in cases treated early, yet in this class of cases I have also had good results without this procedure. The curative effect of this measure, therefore, is still uncertain as far as I am concerned, especially as I invariably employ in addition all the mechanical measures so necessary for success. I have always been extremely careful to secure the most absolute aseptic condition of person and instrument, yet suppuration has followed in a number of instances ; probably it would have occurred under any form of treatment. Rise of temperature, pain, etc., have arisen so speedily that I cannot but feel that the disturbances have been due to the injection. The objections are that it is painful, and is a source of great alarm and distress to children. Thus far I have had no cases of iodoform poisoning, but a certain percentage will doubtless occur.

V.—Removal of Late Deformity.

I.—Tenotomy.
2.—Forcible Straightening.
3.—Osteotomy.
4.—Wedged Shaped Excision.

1.—Tenotomy.

For the correction of flexion without anchylosis, even with slight posterior displacement, tenotomy of the ham string tendons is the best operation in the young, since if accompanied with subsequent fixation with gypsum, it will in the future give an excellent limb, often without anchylosis. Opportunity is thus given for the lengthening of the bones by growth. In performing tenotomy care should be taken to avoid the popliteal vessels and the peroneal nerve. Whe there is thickening or contraction of the connective tissue in the popliteal space, an open incision is desirable rather than a subcutaneous one, as much less risk is thereby incurred. With asepsis, good results can be obtained in nearly every case. Few surgeons feel safe in cutting blindly in the popliteal region, and with improved methods of technique the open plan will, in chronic cases, be more satisfactory.

2.—Forcible Straightening.

Forcible extension should be applied with great caution in the majority of cases, even if there is false anchylosis, lest posterior displacement be produced, or inflammatory symptoms be aroused. Manual extension is by far the safest plan, and should be performed after subcutaneous or open section of the ham strings. The contracted tissues should always be slowly stretched and torn until the limb can be brought to a straight position, where it should be retained with a fixed dressing of gypsum.

Forcible instrumental replacement is also very effective.

The cases best suited for operation are those in which indications of tubercular infiltration and thickening about the articulation are minor in degree. In cases where there is much tuberculous deposit it is very dangerous to arouse fresh inflammatory conditions lest rapid degeneration and suppuration take place.

I have recently operated upon a number of the first described cases in this manner with unusually good results without pain or inflammation, and with subsequent anchylosis, thus securing an excellent walking position. This result I largely attribute to the absolue immobility secured with plaster of Paris, and to retain this immobility until anchylosis has taken place; then compelling the patient to walk upon crutches from six to nine months afterward, and then fixing the knee joint by apparatus for a year or two to prevent relapse.

3.—Osteotomy.

When there is anchylosis with deformity at a right angle, osteotomy is not advisable, since the angle at which the fragments of the femur are placed, is so great that it is not likely to be a useful weight bearing member. Excision in this case is the better operation. If the angle of anchylosis is moderate, however, osteotomy of the femur above the condyles may be performed with advantage. Rectification of the deformity and subsequent fixation with gypsum until thorough union is established, will give a useful limb.

It is well in these cases, as in all other operations for the rectification of knee joint deformity, to prevent walking for a long period of time, and to protect the joint from traumatism for a number of months afterward, as relapse is very common. Osteotomy is certain to give good results without suppuration if careful cleanliness is enforced.

4.—Excision.

Excision, more or less wedged shaped, is indicated in right angle deformity in all cases where the deposit about the knee is great and the anchylosis bony. The method of making the section will vary with the surgeon's ideas, and his eye must decide upon which section will bring the bone to a degree of very slight flexion rather than than of hyper-extension. In regard to fixation of the fragments, the same remarks will hold good, as in cases of ordinary incision. If thoroughly and cleanly performed, immediate closure and fixation can be secured. An Esmarch bandage assists in the rapidity of the operation, and its early removal will permit the oozing to cease before the wound is closed.

VI.—Backward Displacement.

I know of no mechanical method for the gradual relief of backward displacement which is at all satisfactory. Such a condition necessarily indicates that the ligaments have been more or less destroyed, and as the posterior thigh muscles are stronger than the anterior, besides being placed in much better position for mechanical action upon the bones, slow yielding in this direction must occur. I have seen numerous and most elaborately constructed splints, which were models from a mechanical point of view, but which were absolutely useless as far as any effect upon the bones was concerned, since the tissues in the polliteal space becomes so sensitive under even slight continued pressure that little good can be accomplished.

Occasionally double extension, long continued, will partially draw the leg forward upon the femur. Traction should be made in the flexed position upon the lower leg with adhesive strips and weight and pulley, while a well padded band passes behind the head of the tibia and is attached to a cord passing over a ring in the wall or ceiling, with weight adjusted carefully to the endurance of the tissues, to draw the displaced tibia forward. It has succeeded to only a slight degree.

Wedged shaped excisions, with section of the ligaments, answers best, but no more bone must be sacrificed beyond the actual amount of diseased tissue that is just sufficient to relieve the deformity.

Conclusions.

I.—Mechanical treatment by rest, fixation and crutches, either axillary, perineal or ischiatic, is absolutely essential both before and after operation.

II.—In children under twelve years of age conservative measures should be carried to the extreme, and all operative procedures should tend to non-interferecne of the epiphyseal line for as long a period as possible. In these young cases, therefore, tenotomy with subsequent fixation should be the primary procedure to be followed by erasion, when necessary, and by excision only when life is absolutely threatened.

III.—In children from ten to fifteen years of age, conservatism should still be the rule, although the dangers from a shortened limb subsequent to operation are not so serious after growth is completed. In adults operative procedures should be early but more radical in character, erasion still being preferable to excision, except in serious degenerative cases.

IV.—Amputation in children should be employed as a dernier resort. In adults with extensive disease, it is often a wise procedure.

V.—The injection of anti-bacillary substances, both extra and intra articular, offers hope of relief ; but this method is yet in its experimental stage.

VI.—After the subsidence of all inflammation the late deformity should be overcome, first by simple measures, as tenotomy with forcible replacement, excision, rarely by osteotomy.

